# *H3F3B* p.K27I-mutant diffuse midline glioma is a distinct subtype of H3K27-altered diffuse midline glioma

**DOI:** 10.1186/s40478-025-02101-0

**Published:** 2025-08-23

**Authors:** Lei Cheng, Min Zhou, Tao Luo, Rongfang Dong, Ni Chen, Xinyong Cai, Xingwen Wang, Hao Wu, Zan Chen, Zuowei Wang, Xueling Qi, Dehong Lu, Lianghong Teng, Fengzeng Jian, Leiming Wang

**Affiliations:** 1 Department of Neurosurgery, Xuanwu Hospital, International Neuroscience Institute, Capital Medical University, #45 Changchun Street, Western District, Beijing, 100053 China; 2 Department of Pathology, Xuanwu Hospital, Capital Medical University, #45 Changchun Street, Western District, Beijing, 100053 China; 3 Department of Pathology, Sanbo Brain Hospital, Capital Medical University, Beijing, 100093 China; 4 Institute of Pathology and Southwest Cancer Center, Southwest Hospital, Third Military Medical University (Army Medical University), Chongqing, 400038 China; 5 Beijing Jishuitan Hospital, Capital Medical University, Beijing, 100035 China; 6 Laboratory of Pathology, West China Hospital, Sichuan University, Chengdu, Sichuan China; 7 Buping Medical Laboratory Co. Ltd (BP Health), Hangzhou, China

**Keywords:** Diffuse midline glioma, H3F3B, Histone H3, Methylation, DNA mutation

## Abstract

H3K27-altered diffuse midline glioma (DMG) is a fatal disease, including four subtypes H3.3-mutant, H3.1/H3.2-mutant, H3-wildtype with EZHIP overexpression, and *EGFR*-mutant. *H3F3B*, another gene encoding histone H3.3 in addition to *H3F3A*, was ever reported to be mutated in DMGs. However, the clinical and molecular characteristics of *H3F3B*-mutant DMGs is yet understood. The clinical and radiological information of 9 patients with *H3F3B*-mutant DMG were retrospectively collected. Tumor specimens underwent DNA methylation profiling and next-generation sequencing. All tumors harbored somatic *H3F3B* p.K27I mutation. Average patient age was 46 ± 6.86 years, 6 tumors located in spinal cord, 5 tumors involved brainstem and 2 arose in the thalamus. Immunohistochemistry showed these tumors exhibited completely or mosaic-like loss of H3K27me3 expression. Unsupervised t-distributed stochastic neighbor embedding (t-SNE) analysis of DNA methylation profiles showed that *H3F3B*-mutant DMGs formed a unique methylation cluster separate from other gliomas with H3K27me3 loss and DMGs with canonical histone H3 mutation. *PPM1D* and *NF1* were frequently mutated in *H3F3B*-mutant DMGs. Survival analysis showed that *H3F3B*-mutant DMGs had poor prognosis comparable to H3K27M-mutant DMGs. Taken together, *H3F3B* mutation also cause a loss of H3K27 trimethylation in DMGs and result in poor prognosis. The distinct characteristics of DNA methylation and mutational spectrum between *H3F3B*-mutant DMGs and canonical H3K27M-mutant DMGs might suggest divergent underlying mechanism of gliomagenesis.

**Supplementary Information:**

The online version contains supplementary material available at 10.1186/s40478-025-02101-0.

## Introduction

Diffuse midline glioma (DMG) harboring canonical H3K27M mutations (H3.3 or H3.1) is a lethal malignancy with a median survival of 9–19 months [1]. In the 2021 WHO classification of central nervous system (CNS) tumors, the nomenclature has been revised to “Diffuse midline glioma, H3K27-altered” to encompass additional pathogenic mechanisms, including EZHIP overexpression and *EGFR* alterations [2–4]. Consequently, this entity comprises four subtypes: H3.3-mutant, H3.1/H3.2-mutant, H3-wildtype with EZHIP overexpression, and *EGFR*-altered. Among the two genes encoding histone H3.3 (*H3F3A* and *H3F3B*), *H3F3A* p.K27M represents the most frequent mutation in H3.3-mutant DMG. In contrast, *H3F3B* mutations are extremely rare in gliomas, with fewer than five reported cases of *H3F3B*-mutant DMG in the literature to date. Owing to the scarcity of *H3F3B* p.K27I mutations, the clinicopathological and genomic characteristics of *H3F3B* p.K27I-mutant DMG remain poorly characterized [5–7]. Therefore, we performed an integrated radiological, clinico-pathological and molecular (including DNA methylation profiling) characterization on a cohort of 13 patients with *H3F3B* p.K27I-mutant DMG aiming to provide further insight into the developmental nature of this tumor subset.

## Methods

### Sample collection

Tumor specimens and corresponding clinical data from the 9 patients were obtained from the consultation archives (2019–2025) of the Department of Pathology, Xuanwu Hospital, Sanbo Brain Hospital, Capital Medical University and The Southwest Hospital of Army Medical University. Initially, the decision to perform *H3F3B* mutation testing for all the 9 cases was prompted by immunohistochemical (IHC) findings demonstrating loss of H3K27me3 expression, negative H3K27M staining, and EZHIP overexpression. Subsequent next generation sequencing (NGS) was performed and confirmed *H3F3B* p.K27I mutation in all cases. Two neuropathologists independently reviewed all tumors according to the WHO 2021 Classification of Tumors of the Central Nervous System [2]. The study was approved by the ethics committee of Xuanwu Hospital, Capital Medical University and was conducted in full compliance with all principles of the Helsinki Declaration. Additionally, clinical information and DNA mutation data of 4 patients with *H3F3B* p.K27I-mutant DMG from previous literatures were incorporated into our study [5–7].

### Immunohistochemistry

Immunohistochemistry (IHC) was performed on 4-micron-thick formalin-fixed, paraffin embedded tissue (FFPE) sections. The utilized primary antibodies were as follows: H3K27M (ABE419; Millipore, Billerica, MA; 1:1000 dilution), H3K27me3 (clone C36B11, Cell Signaling, 1:50 dilution), Olig-2 (AB9610, Millipore, Billerica, MA, USA; 1:250 dilution), GFAP (polyclonal, Dako, USA; 1:1000 dilution), Syn (ZA-0506; Zhongshan Biotechnology Company, China), ATRX (HPA001906, Sigma, polyclonal, 1:100 dilution), EZHIP (HPA004003, Sigma, polyclonal, 1:1000 dilution), p53 (clone DO-7, Dako, Glostrup, Denmark; 1:100 dilution), Ki-67 (MIB-1, Labvision, USA; 1:50 dilution). All staining was performed using Leica Bond automated staining processors.

### NGS analysis

DNA sequencing libraries were generated using GENESEEQ PRIME Solid Tumor Panel according to the manufacturers’ instructions. Libraries were sequenced on an Illumina HiSeq 4000 (Illumina, Inc., San Diego, CA). Sequencing data were processed through a custom bioinformatics pipeline that was developed to detect single-nucleotide polymorphisms and small insertions-deletions (< 50 base pairs).

### DNA methylation profiling data

DNA was extracted using the QIAamp^®^ DNA FFPE Tissue Kit (Qiagen, Hilden, Germany) for FFPE samples and was restored using the Infnium HD FFPE Restore Kit (Illumina, San Diego, California, USA). Bisulfte conversion was performed using the Zymo EZ DNA methylation Kit (Zymo Research, Irvine, California, USA). Standard quality controls confirmed DNA quality/quantity and bisulfte conversion. DNA methylation data was obtained using Illumina Epi_v2 Beadchip array (935 K) (Illumina, San Diego, California, USA) and Methylation microarray scanner (Illumina NextSeq 550). t-Distributed Stochastic Neighbor Embedding (t-SNE) clustering was done with *Rtsne* package (version:0.16).

## Results

### Clinico-radiological characteristics

**Fig. 1:**
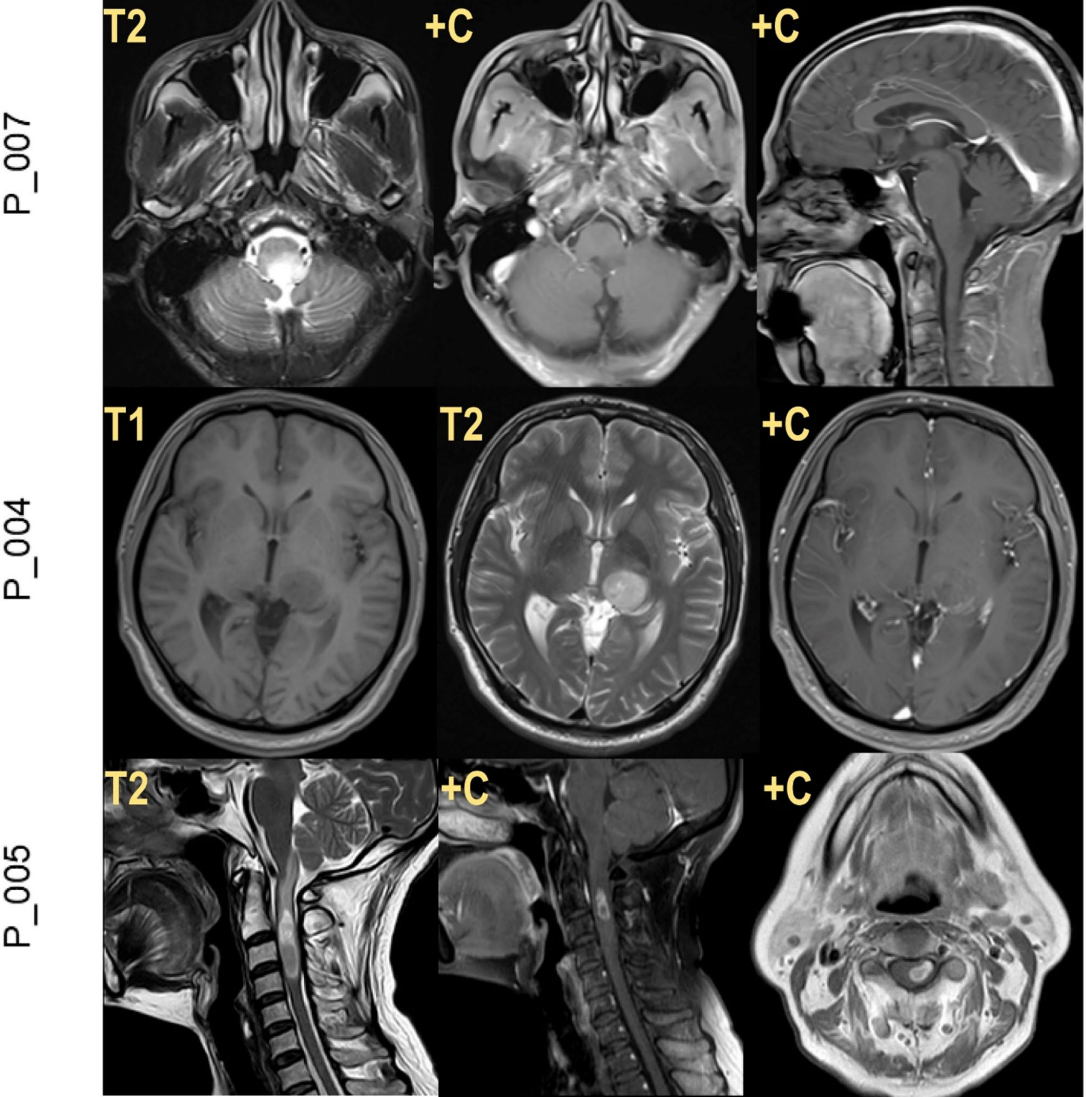
Illustrative MRI of patients with *H3F3B* p.K27I-mutant DMGs

The mean age at diagnosis was 46 ± 6.86 years, significantly older than the average age of patients with H3K27M-mutant DMG [8, 9]. The cohort comprised 9 males and 4 females. In terms of anatomical distribution, 6 tumors located in spinal cord, 5 tumors involved the brainstem (pons: 3, medulla: 2) and 2 occupied the thalamus. Detailed clinical characteristics are summarized in Table 1, with representative MRI findings presented in Fig. 1.

### Histological characteristics

**Fig. 2:**
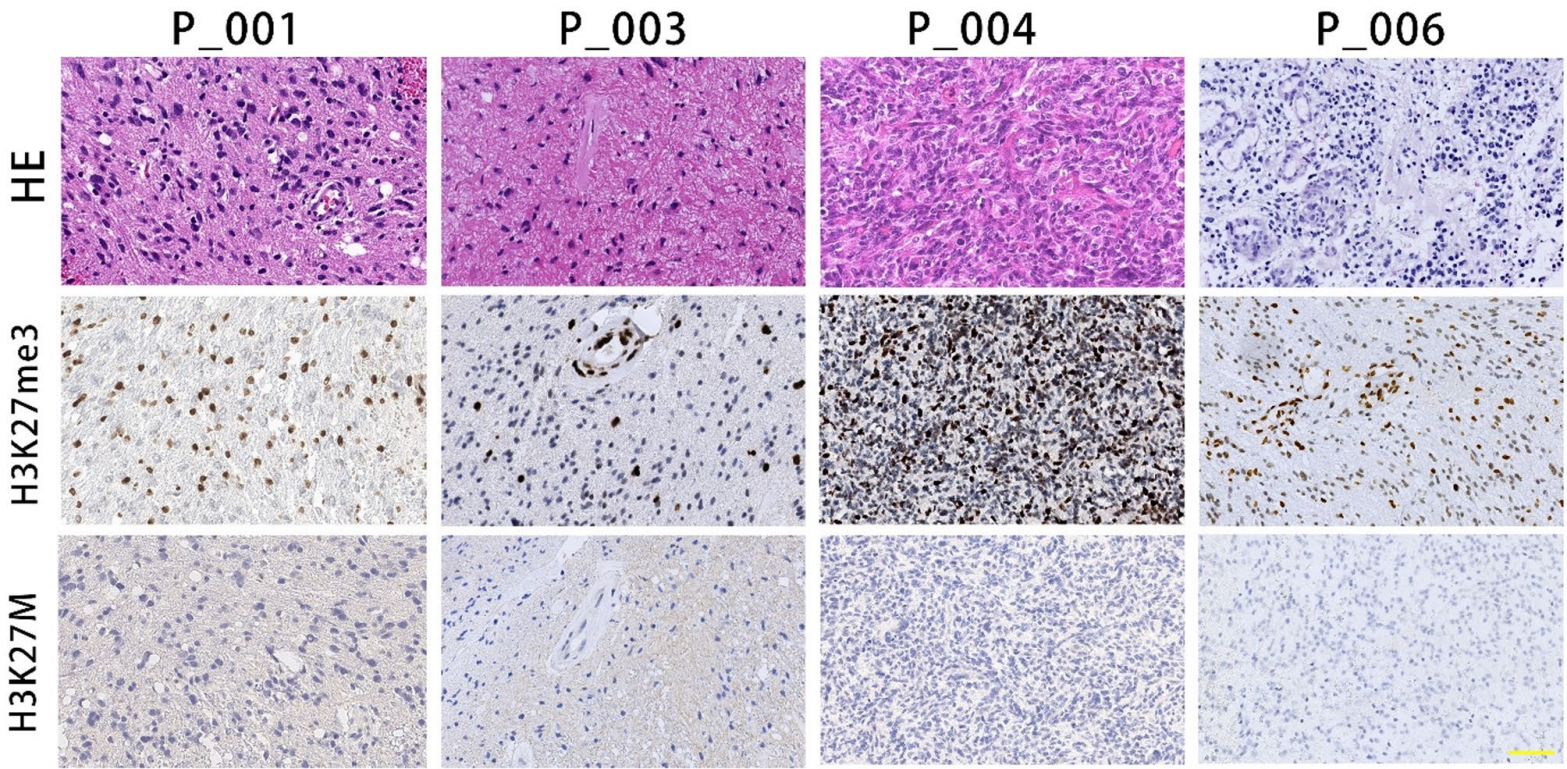
Illustrative histological appearance of *H3F3B* p.K27I-mutant DMGs. All tumors showed completely or mosaic-like H3K27me3 loss and immuno-negative for H3K27M

Histological assessment of *H3F3B* p.K27I-mutant DMGs demonstrated heterogeneous morphology. 3 tumors met criteria for diffuse astrocytoma with moderate cellular density, and absence of mitotic figures, microvascular proliferation, or necrosis. 3 tumors exhibited anaplastic features with elevated cellular density and increased mitotic activity. The other 3 cases showed glioblastoma features with multinucleated tumor cells, microvascular hyperplasia and necrosis. IHC showed that all tumors were immuno-negative for H3K27M. 3 tumors showed mosaic-like loss of H3K27me3 expression (Fig. 2), and the other 6 tumors exhibited loss of expression in the nuclei of tumor cells for H3K27me3. 9 tumors (100%) were diffusely or partially positive for GFAP and Olig-2. 2 (22.2%, 2/9) showed diffusely positive for p53 staining. None of EZHIP (CXorf67) expression or ATRX loss of expression were observed in all 9 tumors (100%, 9/9).

### Distinct methylation pattern between *H3F3B* p.K27I-mutant and canonical H3K27-altered DMG

**Fig. 3:**
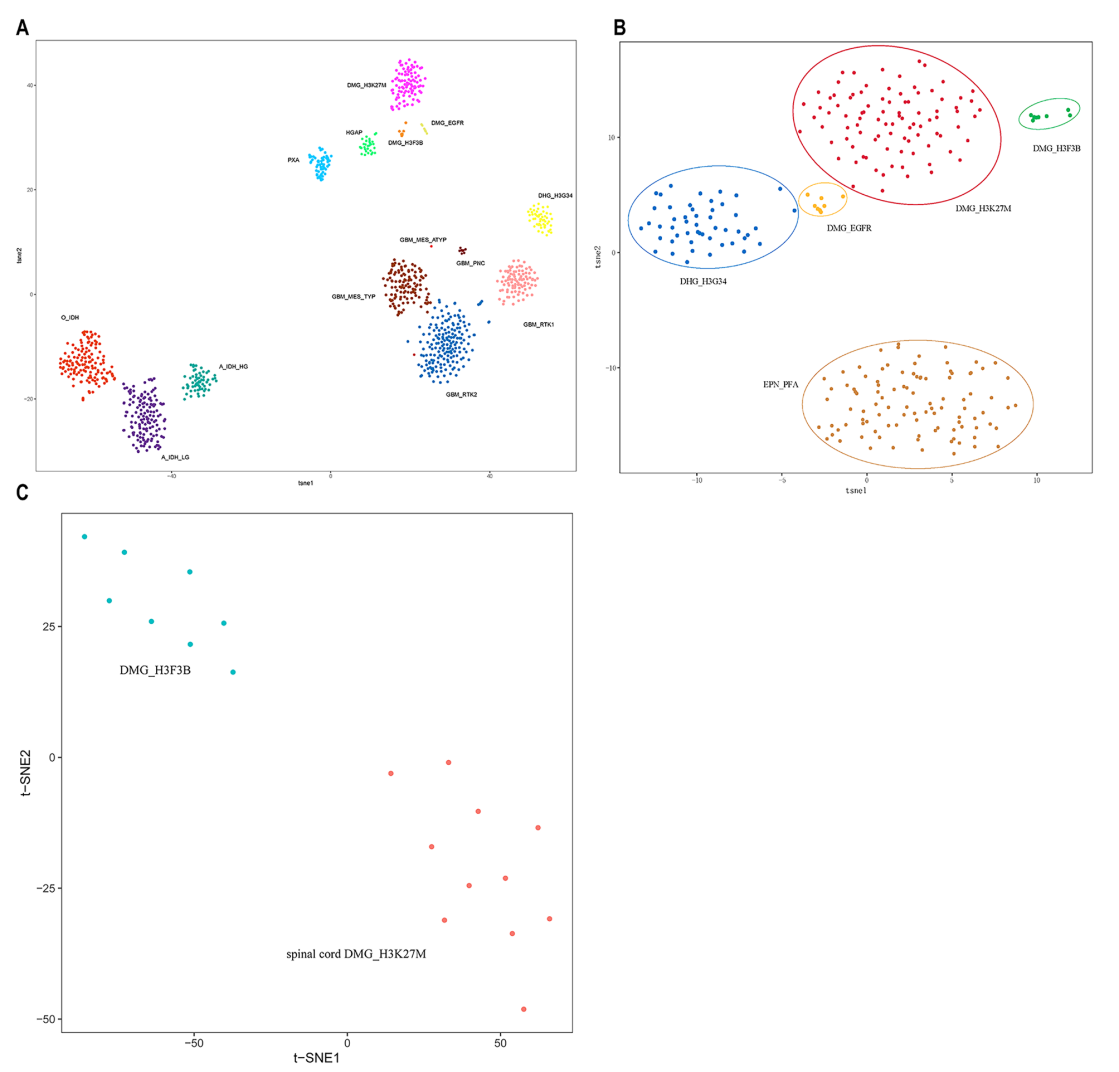
t-SNE clustering analysis of DNA methylation data against 13 reference gliomas (**A**) and against gliomas with histone H3 mutation and gliomas with H3K27me3 loss (**B**) and against spinal cord H3K27M-mutant DMG (**C**) all showed *H3F3B* p.K27I-mutant DMGs formed a unique cluster. **O-IDH**: Oligodendroglioma, IDH-Mutant; **A_IDH_HG**: Astrocytoma, IDH-mutant, High grade; **A_IDH_LG**: Astrocytoma, IDH-mutant, Low grade; **PXA**: Pleomorphic xanthoastrocytoma; **HGAP**: High-grade astrocytoma with piloid features; **GBM_MES_TYP**: Glioblastoma, IDH-wildtype, mesenchymal type; **GBM_MES_ATYP**: Glioblastoma, IDH- wildtype, mesenchymal subtype, atypical; **GBM_PNC**: Glioblastoma, IDH-wildtype, with primitive neuronal component; **GBM_RTK1**: Glioblastoma, IDH-wildtype, RTK1 type; **GBM_RTK2**: Glioblastoma, IDH-wildtype, RTK2 type; **DHG_H3G34**: Diffuse hemispheric glioma, H3 G34-mutant; **DMG_H3K27M**: Diffuse midline glioma, H3 K27M-mutant; **DMG_EGFR**: Diffuse midline glioma, H3 K27-altered, subtype *EGFR*-altered; **DMG_H3F3B**: Diffuse midline glioma, *H3F3B* p.K27I; **EPN_PFA**: Posterior fossa group A ependymoma

Given the distinct H3K27me3 loss pattern between *H3F3B* p.K27I-mutant and canonical H3K27-altered DMG, we performed DNA methylation sequencing to characterize epigenetic divergence. Among eight tumors with available methylation data, t-SNE clustering analysis revealed that *H3F3B* p.K27I-mutant DMGs formed a unique cluster separate from gliomas with H3K27me3 loss and DMGs with canonical histone H3 mutation (Fig. 3A-B and supplementary Fig. 1A). Notably, since tumor location (predominantly spinal cord in our cohort) significantly influences methylation patterns, we included 11 spinal cord H3K27M-mutant DMGs as anatomical controls. Reassuringly, *H3F3B* p.K27I-mutant DMGs maintained a distinct methylation signature compared to location-matched controls, confirming their unique epigenetic landscape (Fig. 3C and supplementary Fig. 2).

### Mutational profiles

**Fig. 4:**
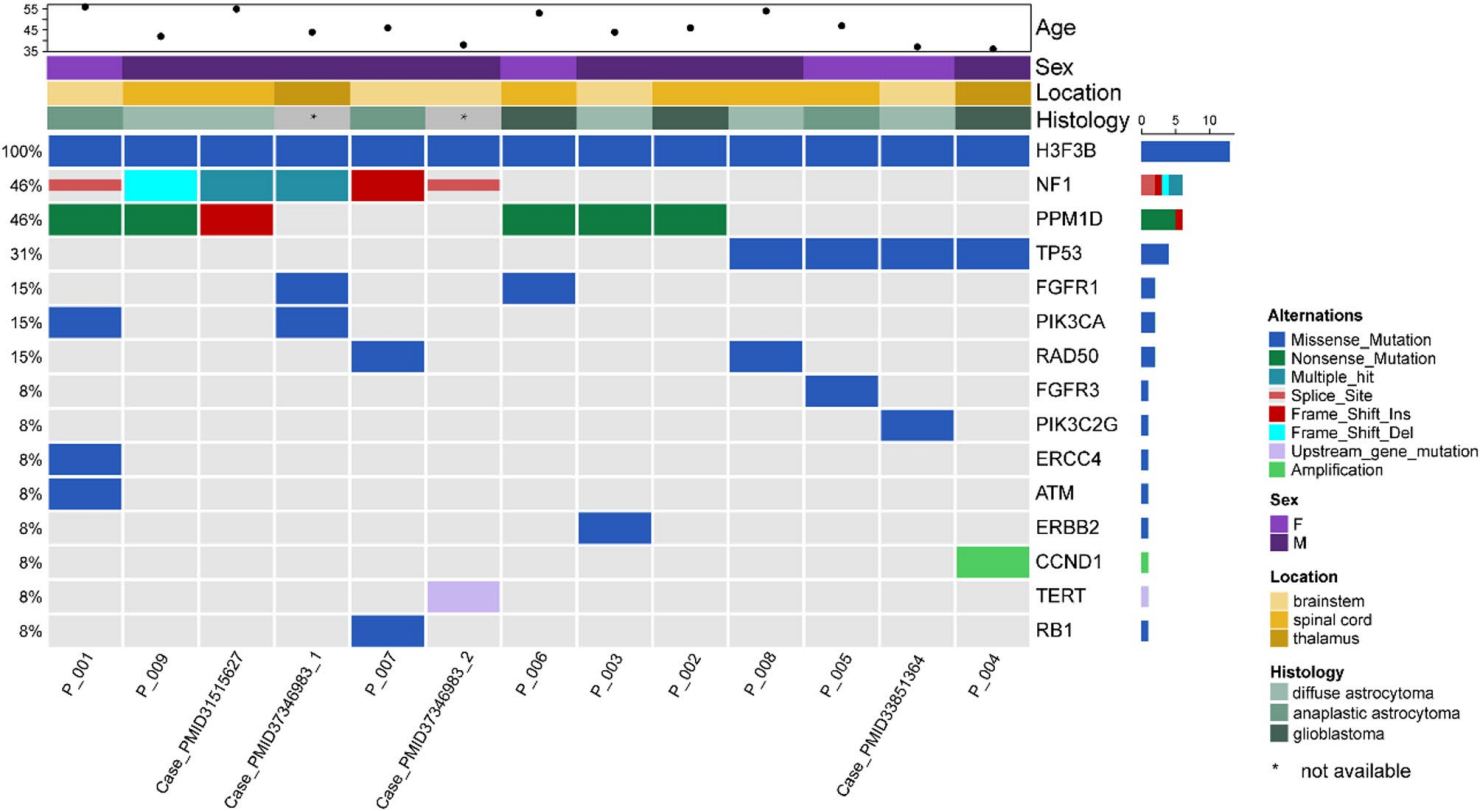
Mutational spectrum of *H3F3B* p.K27I-mutant DMGs. Case_PMID31515627 from study by Sloan EA [6], Case_PMID37346983_1 & Case_PMID37346983_2 from study by Diaz M [5], Case_PMID33851364 from study by Wang Y [7]

Distinct methylation patterns may suggest unique mutational profiles in these tumors. Hence, we performed next-generation sequencing to delineate the mutational profiles of *H3F3B* p.K27I-mutant DMGs. Consistent with their epigenetic divergence, *H3F3B* p.K27I-mutant DMGs exhibited distinctive genomic alterations. Our data showed, besides *H3F3B* p.K27I mutation, *PPM1D* was the most frequently mutated gene (5 cases), and *TP53* mutation was observed in three cases. *NF1* mutation was observed in three cases. No *ATRX* mutation and *MGMT* promoter methylation was found in all cases. Taken together, a total of 6 tumors harbored *PPM1D* and *NF1* mutation, 4 tumors had *TP53* mutation. As compared to the genetic alterations of H3K27M-mutant DMGs [10], significant difference was observed in the prevalence of *PPM1D* mutation (*p* = 0.004) and *NF1* mutation (*p* < 0.001), while the prevalence of *TP53* mutation and *ATRX* mutation did not show significant difference (*p* = 0.169, *p* = 0.226, respectively). Figure 4 showed mutational spectrum of *H3F3B* p.K27I-mutant DMG.

### Survival outcome

**Fig. 5:**
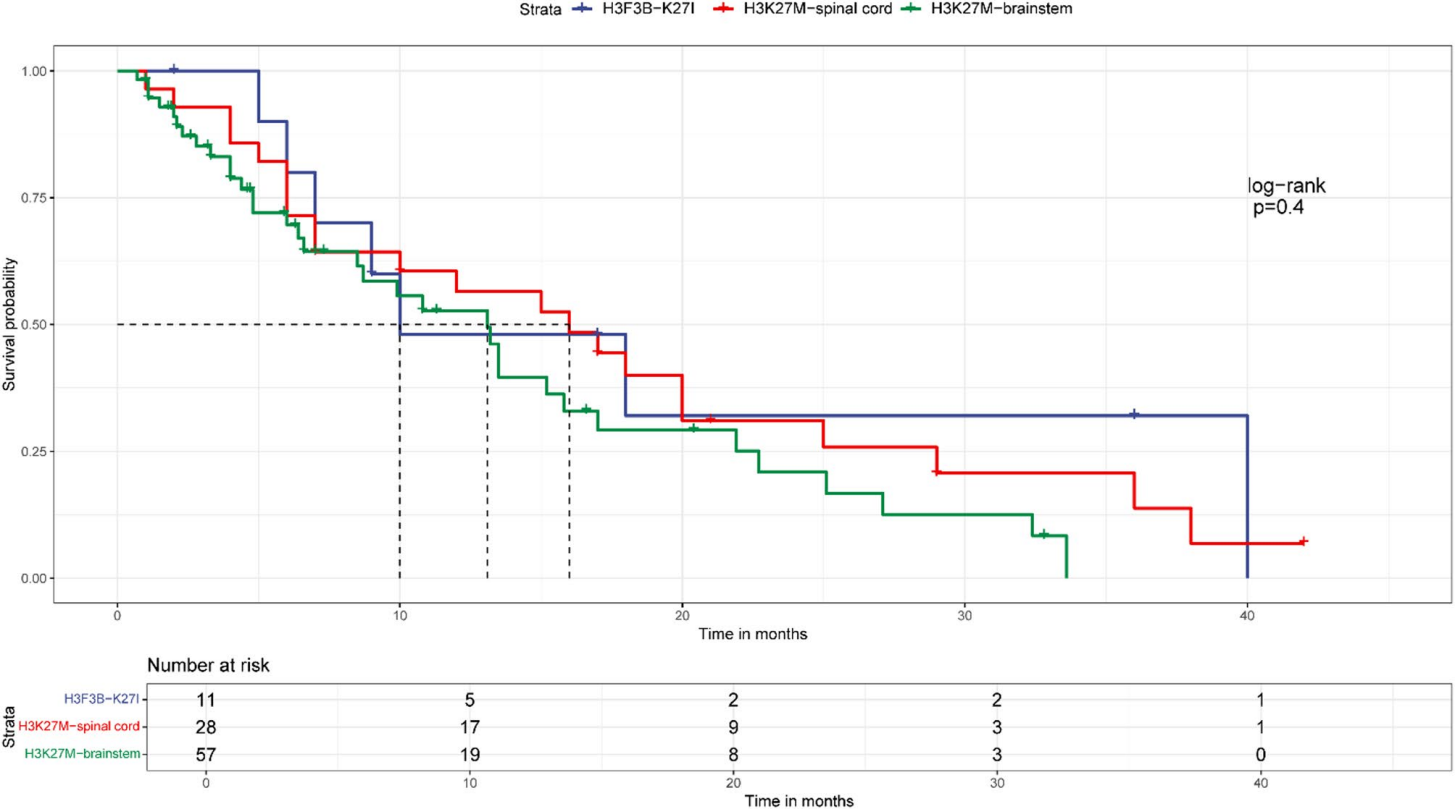
Survival analysis of *H3F3B* p.K27I-mutant DMGs against spinal cord H3K27M-mutant DMGs and brainstem H3K27M-mutant DMGs

With follow-up data available for 11 patients, 4 patients deceased at the last follow-up. Kaplan-Meier analysis demonstrated that patients with *H3F3B* p.K27I-mutant DMG shared similar dismal survival outcome as patients with intramedullary or brainstem H3K27M-mutant DMG (Fig. 5).

## Discussion

Diffuse midline glioma with H3K27-altered mutation is a rare disease with dismal prognosis. While canonical DMG is characterized by histone H3.3 mutations, predominantly *H3F3A* p.K27M, mutations in *H3F3B* (which also encodes H3.3) remain extremely rare [5–7]. To date, the clinicopathological spectrum of *H3F3B*-mutant DMG has been poorly defined. Here, we present the largest cohort of *H3F3B* p.K27I-mutant DMGs, revealing distinct biological features with critical clinical implications.

In our series, all tumors harbored the *H3F3B* p.K27I mutation and arose in CNS midline structures, with spinal cord involvement observed in over half of cases. To our knowledge, 4 *H3F3B* p.K27I-mutant DMGs were previously reported and 2 tumors were located in brainstem, 1 in thalamus and 1 in spinal cord [5–7]. Interestingly, the average age at diagnosis in our cohort is older than that of patients with H3K27M-mutant DMGs [9, 11–15]. Importantly, in our cases, as compared to H3K27M-mutant DMG, *PPM1D* and *NF1* were the most frequently mutated genes, followed by *TP53* mutation, while no *ATRX* mutation was found. Our earlier work identified *TP53* as the most frequently mutated gene in H3K27M-mutant primary spinal cord astrocytoma, followed by *NF1*, *ATRX* and *PPM1D* mutation [16]. Similarly, the study by Chai RC et al. [17] revealed that the most frequently mutated genes included *TP53*, *NF1*, *PDGFRA*, *KIT*, *ATRX* and *RB1 in* H3K27M-mutant spinal cord glioma. For non-spinal cord H3K27M-mutant DMG, the study by Williams EA [8] demonstrated that the most frequent genomic alterations were in *TP53* (64.3%), *ATRX* (43.8%), *NF1*(24.6%), *PIK3CA* (17.7%), *PIK3R1* (7.1%), *FGFR1* (6.4%) and *PDGFRA* (5.6%). Moreover, besides unique mutational profiles of *H3F3B* p.K27I-mutant DMGs, DNA methylation analysis showed *H3F3B* p.K27I-mutant DMGs formed a cluster separate from all other entities with H3K27me3 loss including H3K27M mutant DMGs, *EGFR*-altered DMGs and posterior fossa group A (PFA) ependymoma. Of note, despite divergent molecular profiles, survival analysis indicated comparable poor outcomes between *H3F3B* p.K27I-mutant and H3K27M-mutant DMG patients. The divergent mutational spectrum and epigenetic features but similar survival outcome between *H3F3B* p.K27I-mutant DMG and H3K27M-mutant DMG might suggest conserved and unique molecular drivers underlying the tumorigenesis of *H3F3B* p.K27I-mutant DMGs. Further larger-scale study is warranted to investigate underlying molecular mechanism.

To our knowledge, this is the first study that comprehensively delineate clinico-radiological and molecular landscape of *H3F3B* p.K27I-mutant DMG. Our study provided insight into *H3F3B* p.K27I-mutant DMGs. These findings have significant implications for clinical practice. In cases where immunohistochemistry demonstrates complete or mosaic-like H3K27me3 loss without H3K27M mutation or EZHIP overexpression, subsequent next-generation sequencing in our cohort revealed rare *H3F3B* p.K27I mutations. Thus, gliomas exhibiting H3K27me3 loss with negative H3K27M or EZHIP immunohistochemical results warrant further next-generation sequencing for definitive molecular characterization.

## Conclusion

*H3F3B* p.K27I-mutant DMG is a rare entity and have poor overall survival comparable to H3K27M-mutant DMG. Distinct mutational spectrum and epigenetic features between *H3F3B* p.K27I-mutant DMG and canonical H3K27M-mutant DMG suggest that *H3F3B* p.K27I-mutant DMG is a distinct subtype of H3K27-altered diffuse midline glioma, and larger-scale study is warranted to investigate the unique mechanism of gliomagenesis.

**Table 1:** Clinical characteristics of 13 patients with *H3F3B* p.K27I-mutant DMGs

Patient ID	Age	Gender	Histological morphology	NF1 mutation	PPM1D mutation	TP53 mutation	ATRX mutation	Tumor location	Status	Survival time (month)
P_001	56	Female	Anaplastic astrocytoma	Yes	Yes	No	No	pons	deceased	7
P_002	46	Male	Glioblastoma	No	Yes	No	No	spinal cord	alive	36
P_003	44	Male	Diffuse astrocytoma	No	Yes	No	No	pons	alive	17
P_004	36	Male	Glioblastoma	No	No	Yes	No	thalamus	deceased	18
P_005	47	Female	Anaplastic astrocytoma	No	No	Yes	No	spinal cord	deceased	9
P_006	53	Female	Glioblastoma	No	Yes	No	No	spinal cord	deceased	40
P_007	46	Male	Anaplastic astrocytoma	Yes	No	No	No	medulla	alive	6
P_008	54	Male	Diffuse astrocytoma	No	No	Yes	No	spinal cord	alive	10
P_009	42	Male	Diffuse astrocytoma	Yes	Yes	No	No	spinal cord	alive	5
*Case_PMID 31,515,627	55	Male	Diffuse astrocytoma	Yes	Yes	No	No	spinal cord	alive	2
*Case_PMID 37346983_1	44	Male	Not available	Yes	No	No	No	medulla	Not available	Not available
*Case_PMID 37346983_2	38	Male	Not available	Yes	No	No	No	thalamus	Not available	Not available
*Case_PMID 33,851,364	37	Female	Diffuse astrocytoma	No	No	Yes	No	pons	alive	9

* cases with *H3F3B* p.K27I-mutant DMG from previous literatures

## Supplementary Information

Below is the link to the electronic supplementary material.

Supplementary Material 1: **Supplementary Fig. 1**. Methylation clustering heatmap of the 20,000 most differentially CpG sites corresponding to Fig. 3B

Supplementary Material 2: **Supplementary Fig. 2**. Methylation clustering heatmap of the 20,000 most differentially CpG sites corresponding to Fig. 3C

## Data Availability

The data that support the findings of this study are available from the corresponding author upon reasonable request.
