# Supplementary figures and images for: H3F3B p.K27I-mutant diffuse midline glioma is a distinct subtype of H3K27-altered diffuse midline glioma
Source: Acta Neuropathol Commun. 2025 Aug 23;13:183. doi: 10.1186/s40478-025-02101-0 (PMC12374277; doi:10.1186/s40478-025-02101-0)

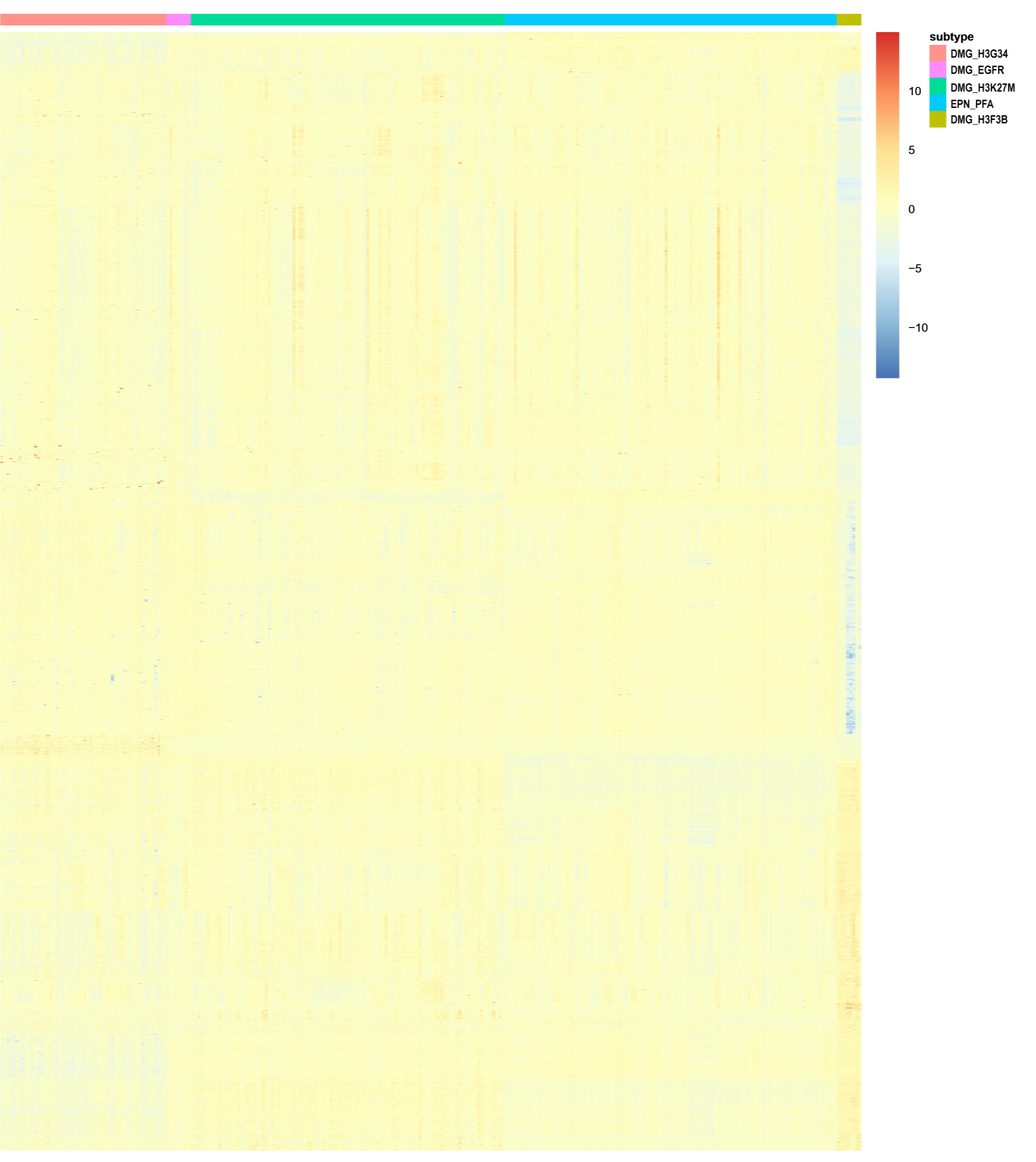

Supplement: Supplementary file 1 — Supplementary Material 1: Supplementary Fig. 1. Methylation clustering heatmap of the 20,000 most differentially CpG sites corresponding to Fig. 3B [file 40478_2025_2101_MOESM1_ESM.tif]

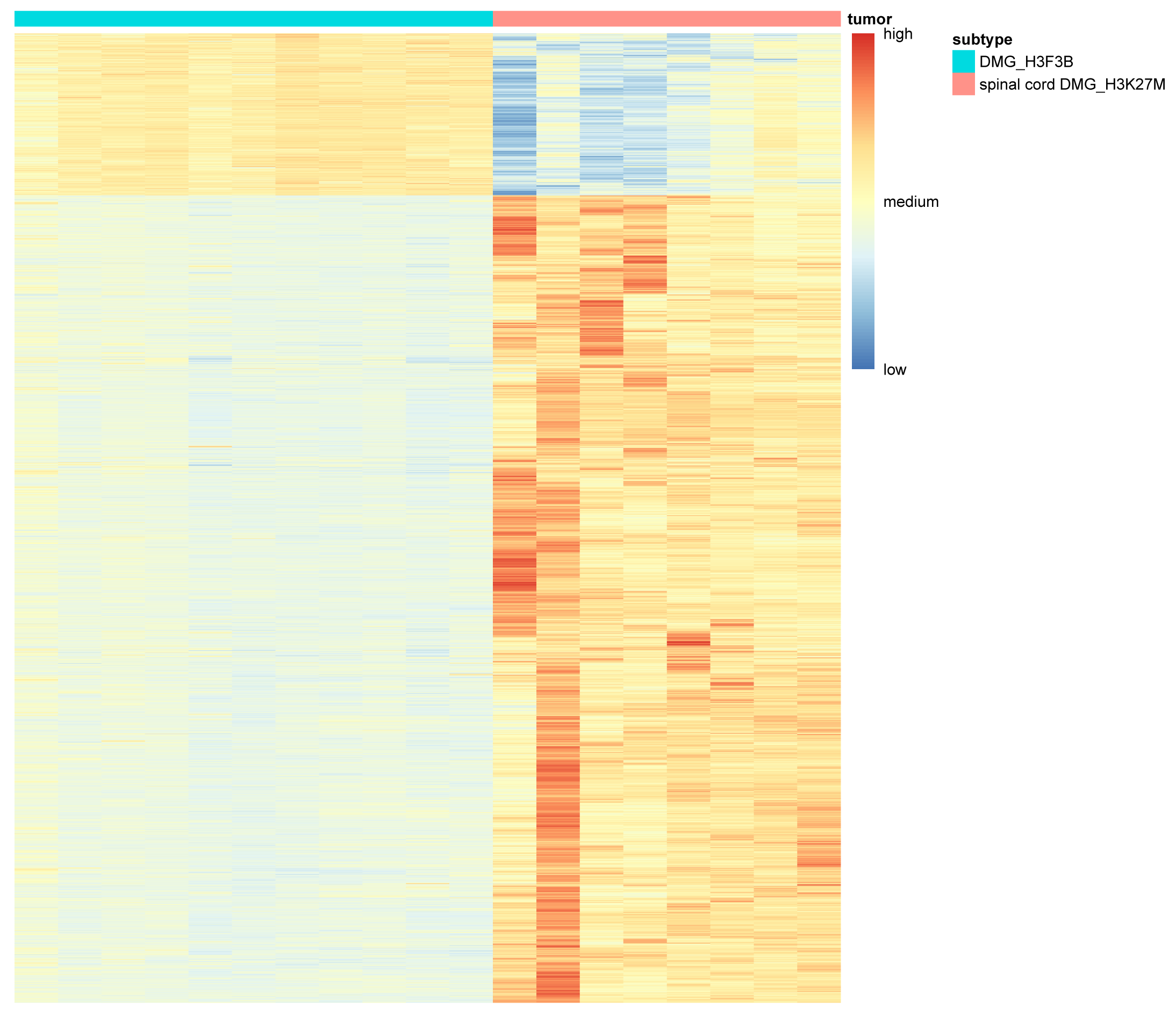

Supplement: Supplementary file 2 — Supplementary Material 2: Supplementary Fig. 2. Methylation clustering heatmap of the 20,000 most differentially CpG sites corresponding to Fig. 3C [file 40478_2025_2101_MOESM2_ESM.tif]
